# High Prevalence of Co-Infections by Invasive and Non-Invasive *Chlamydia trachomatis* Genotypes during the Lymphogranuloma Venereum Outbreak in Spain

**DOI:** 10.1371/journal.pone.0126145

**Published:** 2015-05-12

**Authors:** Mario Rodriguez-Dominguez, Jose Maria Gonzalez-Alba, Teresa Puerta, Blanca Menendez, Ana Maria Sanchez-Diaz, Rafael Canton, Jorge del Romero, Juan Carlos Galan

**Affiliations:** 1 Servicio de Microbiología Hospital Universitario Ramón y Cajal and Instituto Ramón y Cajal de Investigación Sanitaria (IRYCIS), Madrid, Spain; 2 Red Española de Investigación en Patología Infecciosa (REIPI), Madrid, Spain; 3 CIBER en Epidemiología y Salud Pública (CIBERESP), Madrid, Spain; 4 Centro Sanitario Sandoval, Madrid, Spain; 5 Instituto de Investigación Sanitaria Hospital Clínico San Carlos, Madrid, Spain; 6 Laboratorio de Microbiología, Centro Sandoval, Madrid, Spain; 7 Unidad de Resistencia a Antibióticos y Virulencia Bacteriana (RYC-CSIC), Madrid, Spain; University of California, San Francisco, University of California, Berkeley, and the Children's Hospital Oakland Research Institute, UNITED STATES

## Abstract

The evolution of *Chlamydia trachomatis* is mainly driven by recombination events. This fact can be fuelled by the coincidence in several European regions of the high prevalence of non-invasive urogenital genotypes and lymphogranuloma venereum (LGV) outbreaks. This scenario could modify the local epidemiology and favor the selection of new *C*. *trachomatis* variants. Quantifying the prevalence of co-infection could help to predict the potential risk in the selection of new variants with unpredictable results in pathogenesis or transmissibility. In the 2009-2013 period, 287 clinical samples with demonstrated presence of *C*. *trachomatis* were selected. They were divided in two groups. The first group was constituted by 137 samples with *C*. *trachomatis* of the LGV genotypes, and the second by the remaining 150 samples in which the presence of LGV genotypes was previously excluded. They were analyzed to detect the simultaneous presence of non-LGV genotypes based on *pmpH* and *ompA* genes. In the first group, co-infections were detected in 10.9% of the cases whereas in the second group the prevalence was 14.6%, which is the highest percentage ever described among European countries. Moreover, bioinformatic analyses suggested the presence among men who have sex with men of a *pmpH*-recombinant variant, similar to strains described in Seattle in 2002. This variant was the result of genetic exchange between genotypes belonging to LGV and members of G-genotype. Sequencing of other genes, phylogenetically related to pathotype, confirmed that the putative recombinant found in Madrid could have a common origin with the strains described in Seattle. Countries with a high prevalence of co-infections and high migration flows should enhance surveillance programs in at least their vulnerable population.

## Introduction

Both ECDC and CDC have described a dramatic increase in the rate of *Chlamydia trachomatis* infections reaching 39% in Europe and 20.8% in the US in the 2005–2008 period [1, 2]. The latest reports are confirming these high infection rates in Europe and a slight but continuous increase in the US [3]. Moreover, since 2003, epidemic outbreaks of lymphogranuloma venereum (LGV) have crossed through Europe, particularly in the UK, The Netherlands and Spain [4, 5, 6], however in the US there have been only sporadic cases [7]. This new epidemiological scenario observed in Europe, characterized by a high incidence of genital infections caused by *C*. *trachomatis* together with the global spread of LGV could depict an adequate scenario for genetic exchange between prevalent non-invasive urogenital *C*. *trachomatis* genotypes (D-K) and LGV (invasive genotype L1–L3). This fact might result in the selection of new variants with more virulence or more transmissibility traits [8]. The importance of genetic exchange in the evolution of *C*. *trachomatis* has been demonstrated by the growing availability of complete genome sequences [9–12] and laboratory assays [13]. These recombinant isolates can acquire biological advantages in host infection, virulence [8], tissue tropism [13], immune evasion, or antibiotic resistance [14].

One factor required for selecting new recombinant variants is the high prevalence of co-infections by different *C*. *trachomatis* genotypes. The prevalence of co-infections caused by different *C*. *trachomatis* genotypes has been described to be lower than 3% in Europe, US and Australia [15–19]. However outside these regions, the co-infections are more prevalent, with percentages of 10% in South-America, 7.5–18.3% in Asia and 21.2% in Africa [20–23]. Moreover, genetic exchange is likely enhanced between strains with the same tissue tropism [24], such as genotypes belonging to lineages E-F with genotypes belonging to lineages G-D [11].

Another factor that could affect the selection of new variants is the number of clinically asymptomatic infections, as multiple rounds of infections and potential co-infections might occur, especially in the vulnerable population belonging to sexual networks [25, 26]. Recently, infections caused by *C*. *trachomatis* strains of the LGV genotype were found to be present in 25–53% of asymptomatic patients in Europe [27, 28]. Rectal LGV infections offer multiple opportunities for genetic exchange with non-invasive urogenital strains such as G genotype, which is the most prominent genotype in rectal specimens in men who have sex with men (MSM) [11, 29], whereas genotype E, which is the most prevalent variant in Spain [30] and other parts of the world [22, 31, 32] is frequently found in cervix of asymptomatic women.

In this study, a double real time PCR protocol was proposed to detect the simultaneous presence of invasive and non-invasive *C*. *trachomatis* strains in the same sample, using *pmpH* gene as a molecular marker [33, 34]. In the course of the study we identified a *pmpH*-recombinant variant, previously detected in Seattle in 2002, circulating in MSM.

## Material and Methods

### Clinical samples

The study was carried out in 287 clinical samples collected between 2009 and 2013, distributed in two groups: i) 137 samples, in which the presence of L2 genotype belonging to LGV had been identified. Epidemiological and clinical data from 94 of these cases were previously published [6] and 43 additional samples were included. Globally, 118/137 samples (86.1%) were from rectal origin, 15/137 (10.9%) from urethra, and 2/137 from cervix and 2/137 were penis ulcers. With respect to sexual behavior 130/137 (94.9%) were MSM, being 97/137 (70.8%) HIV positive. As to origin of the patients 75/137 (54.7%) were Spaniards, 51/173 (37.2%) were born out of Spain and 11/137 (8%) were of unknown origin. ii) 150 samples were characterized by the presence of *C*. *trachomatis*, but with a negative result for LGV genotypes. These samples were from rectum (42%), urethra (28%), cervix (21.3%), pharynx (8%) and one case of penis ulcer. The percentage of men was 70% and 59.0% of these were MSM. Samples were maintained at -70°C until performing this study.

### Ethics statement

All samples were recovered from anonymous patients (no identification card was requested at the time of admission). As a sentinel centre for STD surveillance, the patients are registered under an encrypted number code for follow up. Therefore individuals could not be matched with their samples and their epidemiological and clinical data. Samples and epidemiological data were collected for diagnostic purpose under standard of care protocols of sexually transmitted infection. Informed consent, as approved by our institutional Ethics Committee, was not required. Neither additional samples nor personal data were requested for this study. Moreover genetic analysis was only performed on bacterial isolates. The study was conducted according to the principles expressed in the Declaration of Helsinki and was approved by the Ethics Committee of Ramon y Cajal Hospital (Reference 308–13).

### Detection of invasive and non-invasive C. trachomatis genotypes

The DNA extraction was undertaken using Nuclisens EasyMag (BioMérieux, Inc, Durham, NC, USA). Two real time PCRs per clinical sample were performed in the same region of *pmpH* gene to identify mixed infections caused by LGV and non-LGV genotypes. The first real-time PCR was used in the initial diagnosis of LGV infection [6, 35]. A second real-time PCR was designed to only identify urogenital non-invasive *C*. *trachomatis* genotypes (D-K), excluding those related to trachoma (A-C) (Table A in S1 File). In both cases the probes were designed along the deletion of 36 bp present or absent in invasive or non-invasive *C*. *trachomatis* genotypes respectively. PCR conditions are described in supplementary material.

On the other hand, a fragment of 430 bp in *pmpH* gene was amplified (Table B in S1 File), using primers previously described [6], and amplicons were digested with the restriction enzyme AccI. Three patterns could be distinguished based on the number of restriction sites: i) genotypes I-J and K showing two restriction sites, ii) genotypes D-H with only one restriction site, and iii) LGV genotypes with no restriction sites. *pmpH*-real-time PCR helped us discriminate between LGV and non-LGV genotypes. This approach allowed us to distinguish the non-invasive genotypes between D-H and I-K genotypes. Subsequently 85 *pmpH* genes from single infections representing different AccI restriction patterns were sequenced to confirm this strategy of assignation. The precise allocation of genotypes was performed with PCR based on *ompA* gene. (see later)

### Multiplex-PCR based on *omp*A gene for accurate allocation of non-invasive C. trachomatis genotypes.

As the phylogenetic reconstruction of *pmpH* gene correlates with the tropism, (because from the phylogeny point of view, *pmpH* gene has evolved in parallel with the three disease-causing group of *C*. *trachomatis*), *ompA* gene was used to identify the different genotypes. A fragment of 1,100 bp of *ompA* gene was amplified using the previously described P1 and OMP2 primers [16]. Then, this amplified product was used as DNA target in new PCRs, to discriminate among D, E, F and G genotypes and group H-K, based on five pairs of primers, in variable positions of the *ompA* gene (Table A in S1 File)

### Sequencing and phylogenetic analysis of known genes related to tissue tropism

*PmpH* phylogenetic trees were reconstructed by maximum likelihood using PhyML 3.0, and reference nucleotide sequences of all genotypes available were downloaded from the National Center for Biotechnology database. Bootstrap support >70% was considered significant. Recombination events were analysed using Recombination Detection Program (RDP3 Beta 27). To further confirm the putative recombination events detected in *pmpH* gene, new phylogenetic analyses were performed using the fragments assigned to different genotypes according to the proposed breakpoint position(s). Topologies obtained with each fragment were compared by TREE-PUZZLE v5.2.

The detection of *pmpH-*recombinant variant in several *C*. *trachomatis* isolates from MSM patients drove the sequencing of other genes phylogenetically related to tissue tropism, such as *rs2* (492 nt) and *incE-incF* (645 nt), invasiveness in *tarp* (732 nt), in addition to *ompA* (858 nt), gene under positive selection and homologous recombination, and *pmpH* (412nt). The primers and PCR conditions used for the amplification of these genes are described in Table B in S1 File. New maximum likelihood (ML) phylogenetic trees of concatenated genes were performed using at least one reference sequence available in Genbank. Moreover, dated phylogeny was obtained using a Bayesian MCMC (Markov Chain Monte Carlo) method implemented in BEAST v1.5.74 program, using only the G genotypes described in Seattle in 2002 and the putative recombinant strains found in Madrid during the period of study.

### Nucleotide sequences

All sequences obtained were submitted to Genbank (www.ncbi.nlm.nih.gov/genbank) under accession numbers KM096869—KM096953, KM096954—KM096958, KM096959—KM096963 and KM096964—KM096968.

## Results

### Clinical samples with simultaneous presence of invasive and non-invasive *C*. *trachomatis* genotypes.

One hundred and thirty-seven clinical samples with confirmed presence of LGV genotype were tested for simultaneous presence of non-LGV genotypes using the *pmpH* gene as target. Co-presence of LGV and D-K genotypes was observed in 15/137 (10.9%) clinical samples (Table 1). Nine of them were found in rectal samples, 3/15 in urethral samples and 3/15 in cervical samples. All the rectal and urethral cases with co-infections were found in MSM, and all of them presented with severe symptoms as pain, bleeding and purulent discharge. Cervical cases were observed in two women with mild symptoms (abdominal pain and leucorrhea) and one asymptomatic woman.

**Table 1:** Percentage of co-infections in patients with and without presence of LGV, depending on infection site.

	LGV POSITIVE (15/137)					LGV NEGATIVE (22/150)						
		TOTAL	D	E	>2 nonLGV GENOTYPES	TOTAL	D+E	D+F	E+F	F+G	D+G	>2 nonLGV GENOTYPES
**RECTAL**	^1^ **MSM**	9 (60.0%)	3 (33.3%)	1 (11.1%)	^a^5(55.5%)	7 (31.8%)	4 (57.1%)	2 (25.6%)	1 (0.14%)			
	^2^ **HTX**											
**URETHRAL**	**MSM**	3 (20.0%)		3 (100%)								
	**HTX**					7 (31.8%)	2 (25.6%)	1 (0.14%)	2 (25.6%)	1 (0.14%)	1 (0.14%)	
**CERVIX**		3 (20.0%)		3 (100%)		7 (31.8%)	3 (42.8%)		2 (25.6%)		1 (0.14%)	^b^1(0.14%)
**PHARYNX**						1 (4.5%)			1 (100%)			

The 137 patients with simultaneous presence of genotypes related to LGV are described as “LGV positive”. The 150 patients with diagnosis of *Chlamydia trachomatis* infection excluding genotypes belonging to LGV are described as “LGV negative”.

^a^The cases included in “LGV positive” group with more than 2 non-invasive urogenital genotypes are: D+E (2 cases), E+F (1 case), E+G (1 case) and D+G (1 case).

^b^The case of coinfection caused by more than 2 non-LGV genotypes in the LGV group involved D+G+F genotypes.

Abbreviations:

^1^MSM: Men who have sex with men.

^2^HTX: Heterosexual.

### Discriminating urogenital non-invasive *C*. *trachomatis* genotypes detected in mixed infections with LGV in clinical samples

The genotypes present in the 15 co-infection cases, with simultaneous diagnosis of LGV infection were determined with the multiplex-PCR assay based on *omp*A gene. Genotype E was present in 11/15 samples with ≥2 genotypes, genotype D in 5/15 cases and genotypes F and G were present in one case each and in association with other genotypes (Table 1). It should be noted that in three clinical samples the presence of two non-invasive genotypes was detected. Genotype E was detected in all cases from the urethra and cervix in the heterosexual population, whereas genotype D and co-infections involving ≥2 non-invasive genotypes were recovered exclusively from the rectal samples in MSM (Table 1).

### Detecting mixed infections by ≥2 urogenital non-invasive genotypes of *C*. *trachomatis* in the non-LGV group

One hundred and fifty clinical samples characterized by the presence of *C*. *trachomatis*, but in which LGV was excluded, were selected to determine the prevalence of co-infections caused by ≥2 non-invasive genotypes. According to the AccI-restriction pattern of *pmpH* gene, 14 clinical samples (9.3%) showed a pattern with two restriction sites, in which the presence of variants belonging to genotypes I-J and K was suspected, and the remaining samples belonged to D-H genotypes. Once the *pmpH* gene allowed the first allocation, the genotyping was determined by an *ompA* multiplex-PCR in the remaining 136 clinical samples corresponding to D-H genotypes. This approach allowed us the accurate allocation of genotypes, and also detected the presence of mixed infection based in the simultaneous amplification of *ompA* genes using the different specific primers (as is described in material and methods). In fact, mixed infections were detected in 22 samples (14.6%). Globally, genotype E was the most prevalent genotype detected (57/150, 38%), followed by D (42/150, 28.0%), F (37/150, 24.6%) and G (24/150, 16.0%) among the infections caused by non-invasive *C*. *trachomatis* genotypes. The total amount is higher than 150 as in 22 samples the simultaneous presence of two non-invasive genotypes was observed (E, D, F and G genotypes were present in 15, 15, 11 and 4 cases respectively), the most common association (9/22 cases) being when E+D genotypes were involved. In addition, a triple infection, involving genotypes D, F and G was also detected in an MSM patient (Table 1).

### Phylogenetic reconstruction and identification of recombinant variants

**Fig 1:**
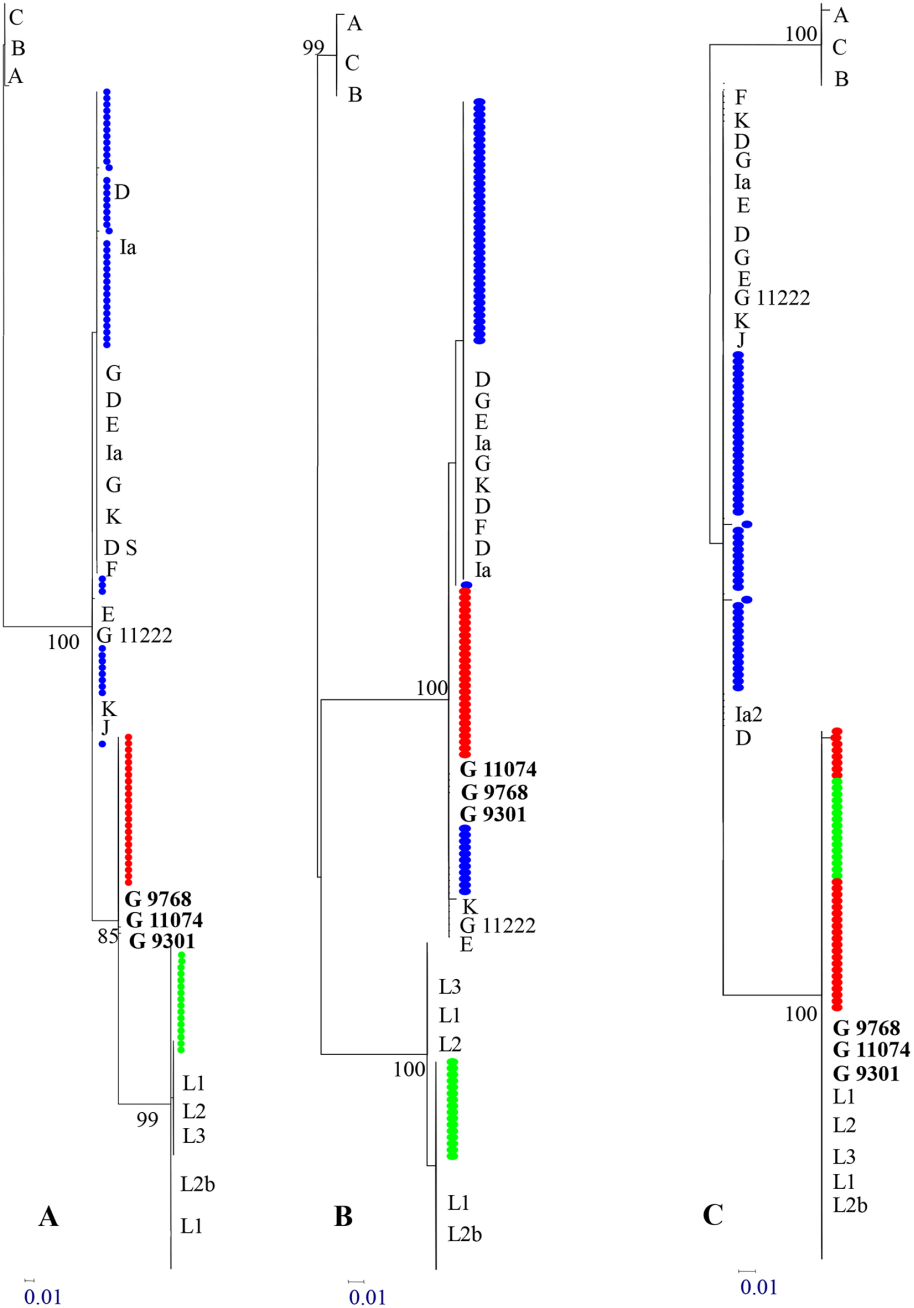
Maximum likelihood phylogenetic reconstruction (PhyML) identifying recombinant variants based on fragment of *pmp*H gene. A) Phylogenetic tree based on complete sequenced fragment of *pmpH* gene (407 bp). B) Phylogenetic tree based on 236 bp (corresponding to 393–555 and 726–800 nucleotides in *pmpH* gene) showing identity to urogenital non-invasive genotypes. C) Phylogenetic tree based on 171 bp (corresponding to 555–726 nucleotides in *pmp*H gene) showing identity to L genotype. The reference strains used in this phylogenetic reconstruction are denominated with the letter which define the genotype. A detailed description of the specific strains used in this tree is shown in Table C in S1 File. In bold we only describe the G-genotypes (G9768, G11074 and G9301) with the highest homology to strains found in Madrid. The colored circles (blue, green and red) correspond to sequences found in Madrid during this work. Green circles correspond to LGV genotypes, blue circles correspond to urogenital non-invasive genotypes (non-LGV) and red circles correspond to putative pmpH-recombinant variant. The evolutionary model used was Hasegawa-Kishino-yano (HKY). Numbers indicate bootstrap values based on 1000 replicates (%).

Eighty five *C*. *trachomatis* strains (25 LGV, 48 D-H and 12 I-K genotypes) from samples carrying only one genotype were selected for sequencing and phylogenetic reconstruction. A complete concordance between the sequencing of *pmpH* genes and the previous assignation, based on restriction site in *pmpH*, was observed. Interestingly, 23 sequences identified as non-invasive urogenital (D-H) genotypes were clustered in a new branch with bootstrap support of 85% (Fig 1A). We suspected that these sequences could be the result of a recombination event. The recombination program detected a putative recombination event in the *pmpH* fragment (393–800 nucleotide position using *C*. *trachomatis* UW-3, belonging to genotype D, as reference sequence) between L genotype (~555–726 nucleotide positions) and a member of urogenital pathotype in the flanking regions (Fig 1B and 1C)

All patients carrying this putative recombinant variant were found in MSM (50% also infected with HIV-1) with minimal symptoms or asymptomatic. With regard to the country of origin, this variant was variably distributed in native (36.6%) and foreign individuals (46.6%), although 16.8% of cases were of unknown origin. It was recovered from the rectum in all cases.

### Molecular and evolutionary characterization of the putative recombinant variant circulating in MSM population

**Fig 2:**
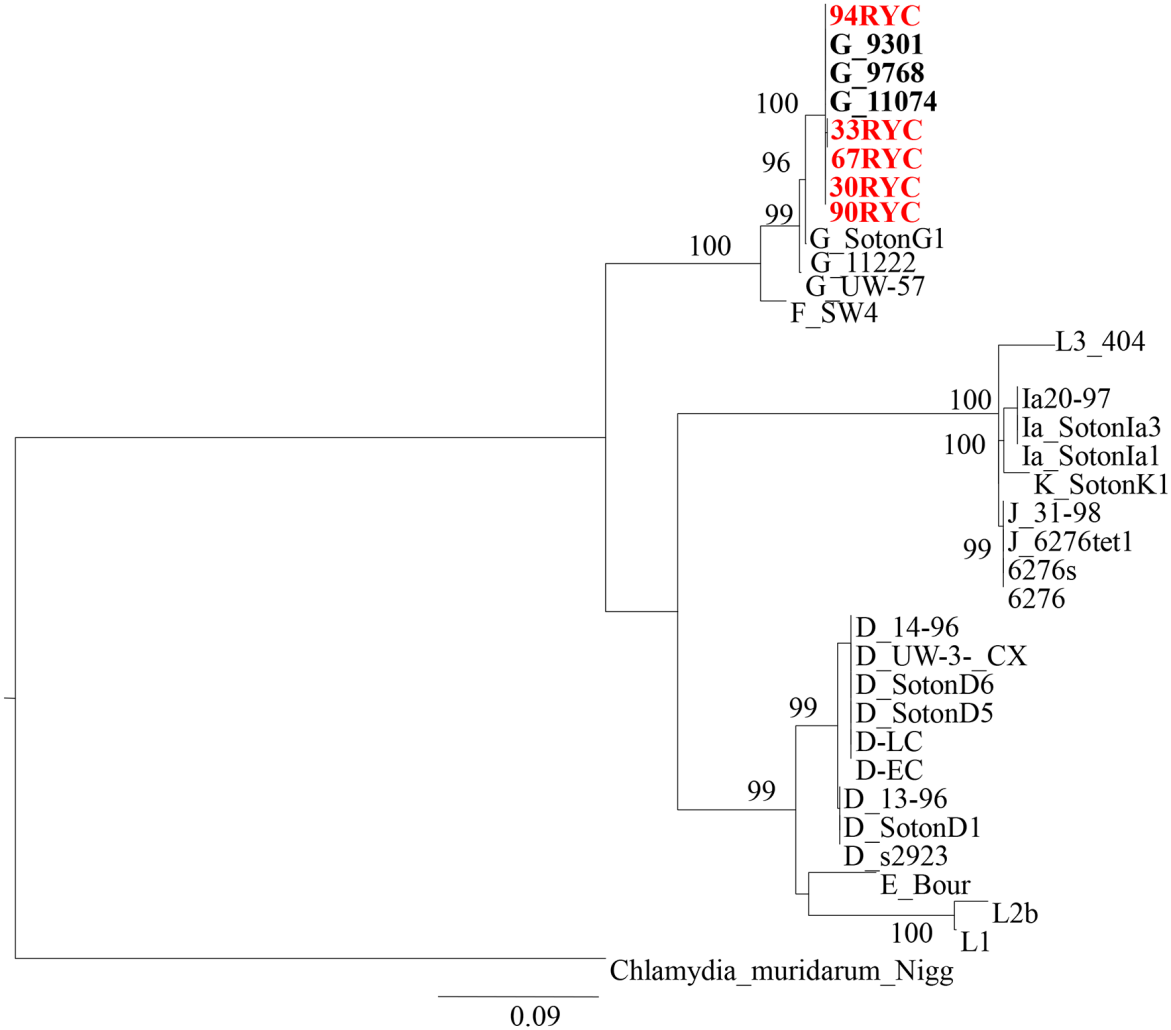
Maximum likelihood phylogenetic reconstruction (PhyML) based on concatenated *pmpH-ompA* genes. The strain carrying the putative pmpH-recombinant variant found during this study (in red letters) is closely related to strains belonging to genotype G (bold). The general time reversible plus proportion of invariable sites and gamma distribution model (GTR+ I+G) was used. Numbers indicate bootstrap values based on 1000 replicates (%).

**Fig 3:**
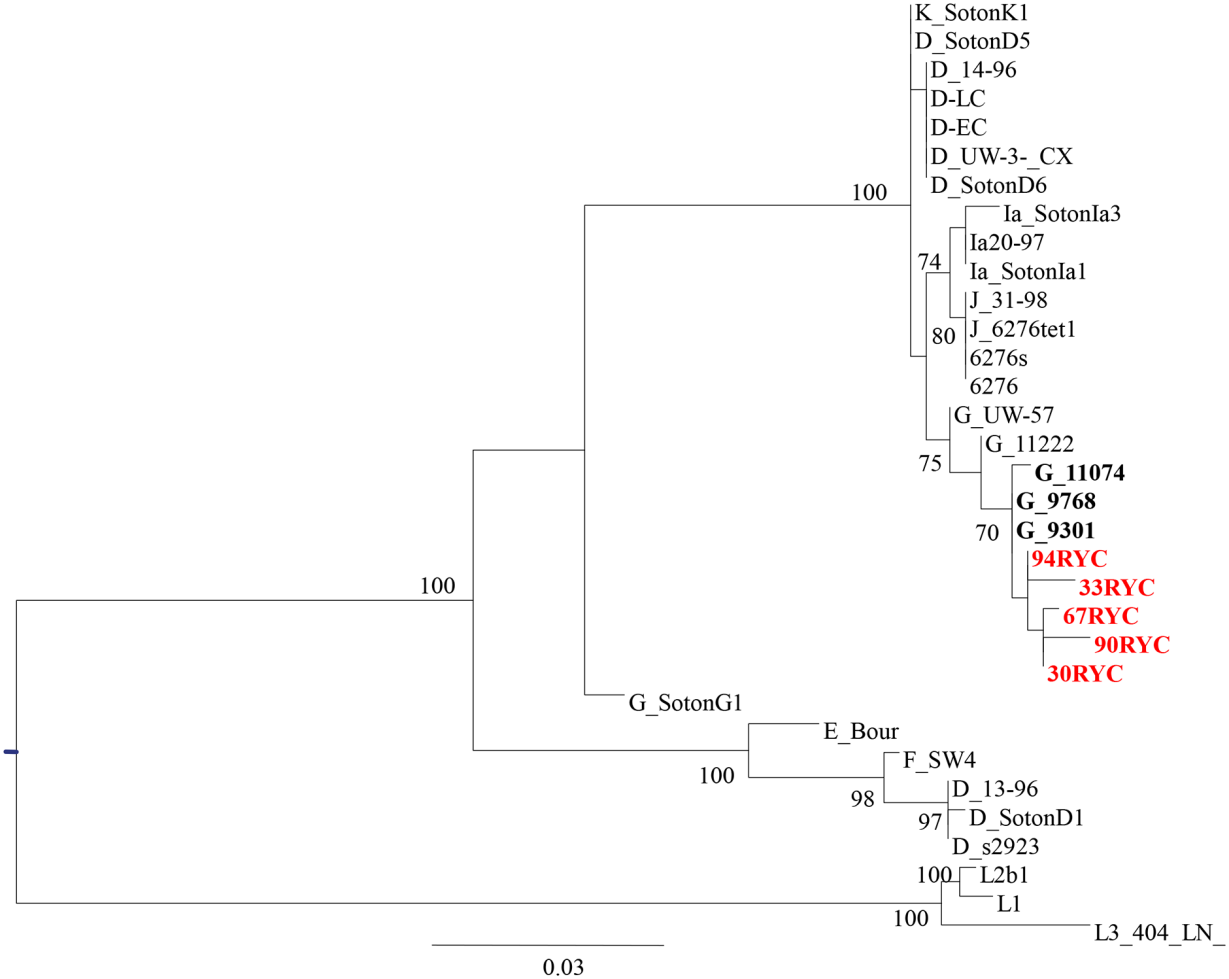
Maximum likelihood analysis of concatenated *rs2-tarP-incEF-pmpH* genes. Red letters correspond to putative pmpH-recombinant variants described in this study. Bold letters correspond to genotype G strains described in Seattle. The general time reversible plus proportion of invariable sites and gamma distribution model (GTR+ I+G) implemented in PhyML software was used. Numbers indicate bootstrap values based on 1000 replicates (%).

**Fig 4:**
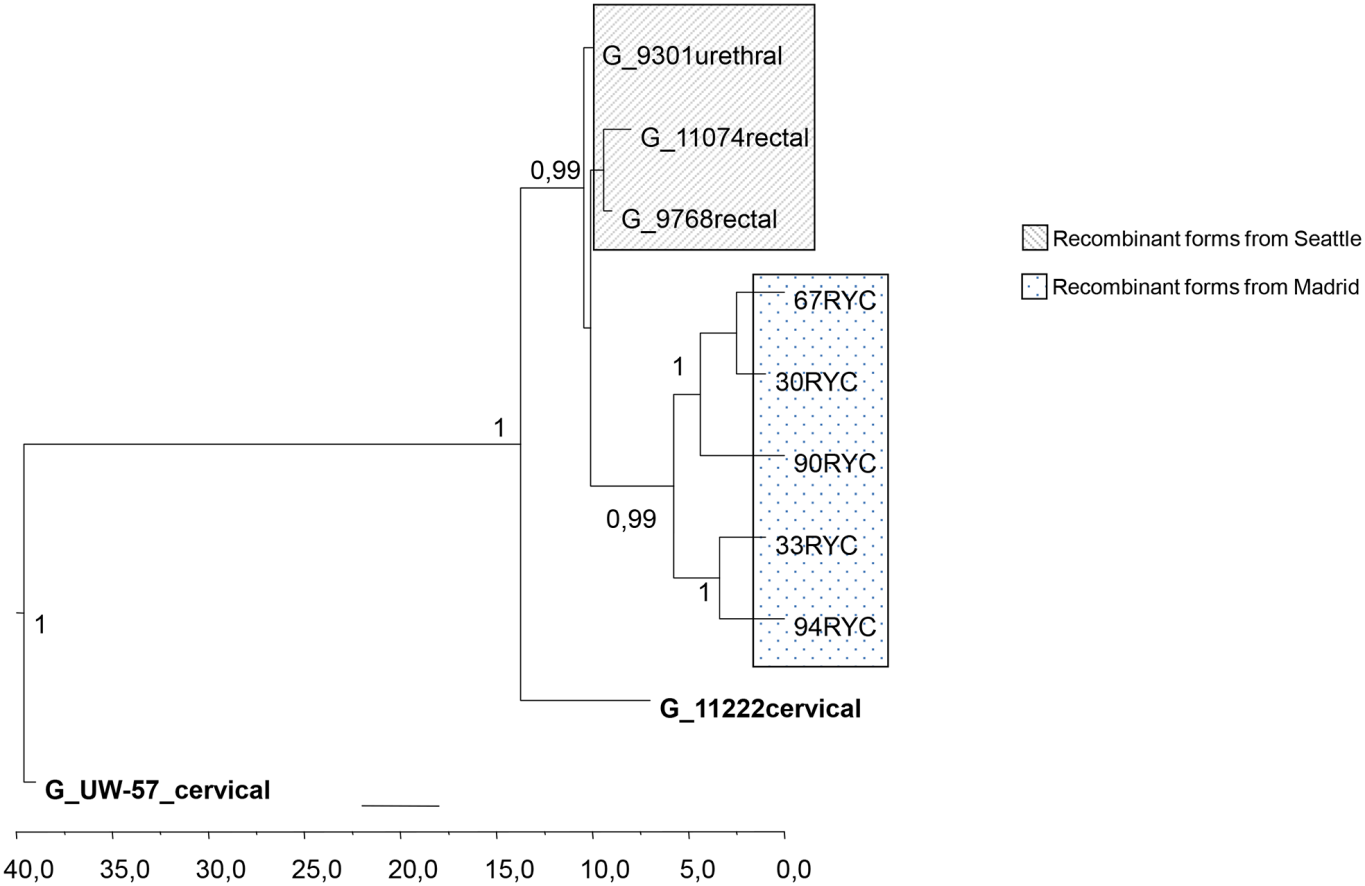
Bayesian analysis (BEAST software) of concatenated *rs2-tarP-incEF-pmpH* genes, using Seattle and Madrid strains belonging to G genotype. The general time reversible plus proportion of invariable sites (GTR+ I) for *rs* gene, Hasegawa-Kishino-Yano, plus proportion of invariable sites (HKY+ I) for *inc* genes and HKY for *tarP* and *pmpH* genes. Numbers indicate posterior probability (>0.9).

As the recombination event was identical in all strains, only five were selected for more complete bioinformatics analysis. A phylogenetic tree based on 1,288 bp of *pmpH-ompA* genes concatenated fragment revealed full identity to a *C*. *trachomatis* variant previously described in Seattle in 2000–2002, initially assigned as genotype G (Fig 2). In order to characterize this *pmp*H-recombinant variant better, we sequenced genes related to pathotypes such as *rs2*, *tarp* and *incE-F* genes, as they can offer good discriminative power (hotspot, insertion/deletions and mutations respectively). The concatenated tree (2,259 bp) showed that the Seattle strains carried an identical *pmpH*-recombinant gene (in order to facilitate the reading, these strains will be refered to as G/LGV-Seattle strain) and the putative recombinant variant found in Madrid constituted a statistically distinguishable monophyletic node (Fig 3). In all selected genes, the highest homology was observed with G/LGV-Seattle strain But in the *rs* gene two G/LGV Madrid strains showed higher homology with D and K genotypes than Seattle recombinant strains. Moreover a G/LGV-Madrid strain had a deletion in the *tarp* gene, which was not present in the G/LGV-Seattle strains. Following the bioinformatics program MEGA 5, the average divergence could be estimated in 4.8 and 0.7 in G/LGV-Madrid strains and G/LGV-Seattle strains respectively, suggesting that G/LGV-Madrid strains could have different evolutionary trajectories (Fig 4)

## Discussion

The evolution of *Chlamydia trachomatis* is mainly driven by recombination and these events are more probable in epidemic situations, because the possibilities of two strains simultaneously infecting the same host are increased. In our study, the percentage of co-infections with two or more urogenital non-invasive *C*. *trachomatis* genotypes was 14.6%. This value, one of the highest published, is more related with developing countries than with a European country [20–24]. The implications for Public Health may be more disturbing in the current epidemiological situation because the co-circulation of LGV strains with non-invasive *C*. *trachomatis* genotypes increases the opportunities of co-infections, especially in vulnerable populations, and *a priori* the results are more unpredictable. A high percentage of mixed infection could facilitate the selection of new recombinant variants, such as the hypervirulent strain described by Sommbonna et al. [8], the result of recombination events between L2 and D genotypes. In our series, the co-infections including LGV and D-K genotypes were 10.9%, the highest prevalence of co-infections described to date [29]. Two limitations must be commented about this point. First, the high data observed correspond to the high-risk population attended in our STI center. For instance, 60% of co-infections caused by invasive and non-invasive genotypes were found in the MSM population. Second, different studies about the prevalence of co-infection in high-risk or general populations have used different molecular techniques, so that the comparative result must be interpreted with caution. These data about the prevalence of co-infection in Spain suggest high opportunities of the emergence and selection of new recombinant variants. In fact as confirmation of this message, a putative *pmpH-*recombinant variant, between L-genotype (555–726 nt) and G genotype, was identified in the MSM population (Fig 1). The description of putative recombinant variants in genes phylogenetically related to pathotypes, such as *pmp* genes, opens a new point of discussion in the diagnosis and typing of *C*. *trachomatis*.

The distribution of genotypes, excluding LGV, in single and mixed infections (128 and 22 respectively) was dominated by E genotype (57 and 15 patients respectively), followed by D (42 and 15 patients), F (37 and 11 patients), G (24 and 4 patients) and finally I-K (14 and 0 patients) genotypes. Apparently, the proportion of genotypes in single and mixed infections is similar; therefore any beneficial association between *C*. *trachomatis* genotypes was excluded. Likewise, the most prevalent genotypes in co-infections including LGV were E (73.3%) and D (33%) (F and G genotypes were found in only one case). However, when considering sexual behavior, the most prevalent genotype in the MSM population was D followed by E (Table 1), but this data is not sufficient to conclude if particular genotypes are circulating in different population groups [36]. Moreover, co-infections characterized by >2 non-invasive genotypes and LGV was only detected in the MSM population. A limitation of our study is the absence of approach for detecting *C*. *trachomatis* genotypes from trachoma.

According to our epidemiological data, the most probable recombinant variant between invasive and non-invasive should have been produced between LGV and D or E genotypes; however the putative recombinant variant identified in our series involved G-genotype. Two reasons, both compatible, could explain the selection of this variant. First, genotype G has been associated with rectal tropism [9] and the current LGV outbreak is associated with proctitis and recovered from rectal samples, increasing the opportunities of genetic exchange between variants belonging to G and LGV genotypes. Second, the origin could be outside Spain. The concatenated *pmpH-ompA* tree (Fig 2) showed identical homology to *C*. *trachomatis* G/9301, G/11074 and G/9768 strains classified as genotype G isolated from urethral and rectum in men in Seattle between 2000–2002 [9]. Phylogenetic analysis of sequenced genes led to the suspicion that the variants from Seattle and Madrid had a common origin. The concatenated tree based on *rs2*, *tarp* and *incE-incF* , confirmed that G/LGV from Seattle and G/LGV from Madrid constituted a statistically distinguishable monophyletic node (Fig 3), suggesting a direct or indirect transmission between both regions. Recently a new LGV variant (characterized by L173F mutation in *omp*A) has been described in the US [7] but this identical *omp*A-variant in LGV was previously described by our group in Madrid [6] suggesting continuous transmission routes between the US and Europe or viceversa. Moreover, among the people with *C*. *trachomatis* infections caused by the putative recombinant variant almost half (46.6%) were found in people born out of Spain. This finding reveals the impact that migration flows could have in the subclinical spreading of new *C*. *trachomatis* variants, especially in receptor countries of immigrants such as Spain [37]. Another suggestive epidemiological aspect in the characterization of this *pmpH*-recombinant variant was the average divergence into the sequences in the G/LGV-Madrid variant in comparison with G/LGV-Seattle. This data suggests that even if G/LGV-Seattle and G/LGV-Madrid strains could have a common evolutionary origin, the strains found in Madrid could have different trajectories (Fig 4). A limitation of this reasoning is the low number of available sequences.

Furthermore, the presence of the highest prevalence of co-infections between invasive and non-invasive genotypes (10.9%) and between non-invasive urogenital variants (14.6%) offers the ideal conditions for the emergence of new recombinant variants. The description of putative recombinant variant between LGV and G-genotype is an example of this risk.

## Supporting Information

S1 File Table A) Primers and probes used in the detection of L- and non-L-genotypes (*pmpH* gene) and generic and specific primers used for genotyping (*ompA* gene). Table B) Primers used for amplify and sequencing the selected genes involved in the characterization of *pmpH*-recombinant variant. Table C) Reference sequences used for phylogenetic analyses.
(DOCX)
