# Supplementary material for: High Prevalence of Co-Infections by Invasive and Non-Invasive Chlamydia trachomatis Genotypes during the Lymphogranuloma Venereum Outbreak in Spain
Source: PLoS One. 2015 May 12;10(5):e0126145. doi: 10.1371/journal.pone.0126145 (PMC4428631; doi:10.1371/journal.pone.0126145)
Supplement: S1 File — Table A) Primers and probes used in the detection of L- and non-L-genotypes (pmpH gene) and generic and specific primers used for genotyping (ompA gene). Table B) Primers used for amplify and sequencing the selected genes involved in the characterization of pmpH-recombinant variant. Table C) Reference sequences used for phylogenetic analyses. (DOCX) [file pone.0126145.s001.docx]

**SUPPLEMENTARY MATERIAL**

Real-time PCR for discriminating between L-genotypes and non-L-genotypes and characterization of non-L genotypes.

The protocol for detection of L-genotypes, belonging to LGV genotypes, was previously described [1, 2] based on 36 pb deletion in *pmp*H gene. In this study, we designed new primers and probe in the same region of *pmp*H gene, using identical reverse primer that PCR described to L-genotypes (cLGV-R, c; common) (Table S1). The mastermix for non-L-genotypes was carried out in a final volume of 25 µL containing 12.5 µL of 2x Taqman Fast PCR Master Mix (Applied Biosystems), 150 nm of each primer (nLGV-F y cLGV-R) and 100 nm of the probe nLGV-PB and 5 µL from eluted DNA. PCR conditions were 95ºC 1 min. followed by 40 cycles of 94ºC for10 s, and 60ºC for 30 s. A final elongation step at 72°C for 10 min was included.

**Table A.- Primers and probes used in the detection of L- and non-L-genotypes (*pmp*H gene) and generic and specific primers used for genotyping (*omp*A gene).**

| **Gene** | **Primers/ probes** | **Sequence (5´-3´)** | **Position** |
| --- | --- | --- | --- |
| *pmp*H | LGV-F | CTGTGCCAACCTCATCATCAA |  |
|  | nLGV-F | CTATTGTGCCAGCATCGACTC | 449-469 |
|  | cLGV-R | AGACCCTTTCCGAGCATCACT | 529-549 |
|  | LGV-PB | FAM-CCGCCTGCTCCAACAGTTAGTGATG-BHQ1 |  |
|  | nLGV-PB | FAM-AGCTCCTGCTGCTTCAAGCTCTTTA-BHQ1 | 492-516 |
|  |  |  |  |
| *omp*A | P1^[3]^ | ATGAAAAAACTCTTGAAATCGG | 1-22 |
|  | OMP2 ^[3]^ | ACTGTAACTGCGTATTTGTCTG | 1103-1124 |
|  | ompADF | AAAACGGTCAAAGCGGAGT | 496-514 |
|  | ompADR | ATCTGTTCCTGCTGTAAGAT | 749-768 |
|  | ompAEF | ACACAGATACTGCCTTCTCT | 557-576 |
|  | ompAER | ATCAGTTCCTGCTATGAGTG | 749-768 |
|  | ompAFF | CACGAAACCTGCTGCAGAT | 498-516 |
|  | ompAFR | GTAATATCTACAACAGGTGTTA | 932-953 |
|  | ompAGF | ACGCAGCCTGCTGCAACA | 499-516 |
|  | ompAGR | GTAATATCTACAACAGGTTTTG | 932-953 |
|  | ompAH-KF | AAACACAATCTTCTARCTTTAATA | 491-514 |
|  | ompAH-KR | TCGTWTCGCTTCCGGAAG | 1001-1019 |

An *omp*A-fragment of 1,100 bp, common to all *C. trachomatis* genotypes, was amplified using the generic primers (P1 and OMP2) described by Jurstrand *et al* [3]. Based on variable sites of *omp*A gene, five couples of primers (Table S1) were designed in order to amplify the genotypes D, E, F, G and group HIJK respectively. All ten primers were designed with the same melting temperature. Then, five independent PCR were performed in a total volume of 50 μL containing 2 μL of the previous generic PCR, reaction buffer (10x), Cl_2_Mg (1,5nM), dNTPs (0,2nM), 0.6 mM of each corresponding primer and 1.5 UI AmpliTaq Gold DNA polymerase (Applied Biosystems, Norwalk, US). PCR conditions were the same in the five PCRs. 94ºC 12 min, followed by 40 cycles of 94ºC/30 s, 57ºC/30 s, and 72ºC/30 s; a final elongation step at 72ºC for 10 min. was performed. Amplified products size varies between 200 to 400 bp, so a subsequent electrophoresis was performed in a 2% agarose gel using 100 bp DNA ladder (Life Technologies Co, California US). When the electrophoresis finished, the gel was stained with Gel Red (Biotium Inc, California, US) during 30 min.

The sequencing of *omp*A and *pmp*H genes revealed high homology with three strains isolated in Seattle (Figure 2). Initially, these strains were assigned as genotype G according to *omp*A gene; however *pmp*H gene was the result of a recombination event between L-genotype and G-genotype. In order to characterize this potential recombinant variant better, we selected several genes correlated to pathotype (*inc*E-F), under positive selection (*tar*p), homologous recombination (*rs*2). These genes are also distributed along the chromosome of *C. trachomatis*. For instance, *inc*E-F (CT-116-CT_117) are located in around nucleotide position 135 Kb (respect to *C. trachomatis* D/UW3 genome annotation), *tar*p (CT_456) in position 53 Kb, *rs2-omp*A (CT_680-CT_681) in 780 Kb and *pmp*H (CT_872) in position 1034 Kb. The nucleotide sequence of primers used to amply these genes are described in table S2

**Table B. Primers used for amplify and sequencing the selected genes involved in the characterization of *pmp*H-recombinant variant**

| **Gene** | **Primer/probe** | **Sequence (5´-3´)** | **Position** |
| --- | --- | --- | --- |
| *inc*E-*inc*F | INC-F | ATGGAATGCGTTAAACAGTTAT | 1-22 1461-1439 |
|  | INC-R | TTTGCAATGCAAAACATAACACC |  |
| ^[4]^*tar*P | CT456-Fm | ACGCAACAAATTCAGCGGCTA | 140-160 1487-1467 |
|  | CT456-RSeq1 | GAACAGACTTGGTCCCAATTT |  |
| ^[5]^*rs*2 | rs2-1 | AGCGCTGCACCACGTTCATCAACT | 153-130  214-190 |
|  | rs2.2 | CGCGCCCGTAGCTCAATGGTAGA |  |
| *omp*A | P1 | ATGAAAAAACTCTTGAAATCGG | 1-22  1103-1124 |
|  | OMP2 | ACTGTAACTGCGTATTTGTCTG |  |
| *pmp*H | pmpH-F | AAACCGTGAGTATTTCCGGA | 380-399 |
|  | pmpH-R | ATATGCCTCCTTCATTGTCTT | 784-764 |

**Amplification and sequencing of *rs*2, *tar*p and *inc*E-*inc*F**

Primers and PCR conditions for amplification and sequencing of *rs*2 and *tar*p were previously described [4, 5]. New primers were designed for *inc*E-*inc*F (Table S2). PCR was performed using AmpliTaq Gold DNA polymerase (Applied Biosystems, Norwalk, US) in a final volume of 50 µL. PCR conditions were 94ºC for 12 min for polymerase activation followed by 40 cycles of 94ºC for 30 s, 1 min. of annealing (at 60ºc for *tarp* and *rs*2 and 57ºc for *inc*E-*inc*F) and extension at 72ºc for 1.5 min. Amplification products were purified with ExoSAP-IT purification kit (USB Corp., Cleveland, OH, US) and sequenced by Sanger method using the same primers in ABI Prism 377 system (PE Norwalk, CONN, US). Amplification and sequencing of *pmp*H was performed using AmpliTaq Gold DNA polymerase 1.5 UI (Applied Biosystems, Norwalk, US) in a final volume of 50 µL containing buffer (10x), Cl_2_Mg (1.5nM), dNTPs (0.2nM), 0.6 mM of each primer. PCR conditions were 94ºC 12 min. for DNA polymerase activation followed by 40 cycles of 94ºC 30s, followed 60ºC /30 s, 59ºC/1 min, and 72ºC/ 1 min, and a final extension step at 72ºC for 10 min. Electrophoresis was carried out in 1% agarose and stained with GelRed 10000X (Biotium Inc, California, US). Amplification products were purified using ExoSAP-IT purification kit (USB Corp., Cleveland, US) and sequenced with the same primers used in amplification.

REFERENCES

1. Morré SA, Spaargaren J, Fennema JS, de Vries HJ, Coutinho RA, *et al*. (2005). Real-time polymerase chain reaction to diagnose lymphogranuloma venereum. Emerg Infect Dis; 11: 1311–1312.
2. Schaeffer A, Henrich B. (2008) Rapid detection of *Chlamydia trachomatis* and typing of the lymphogranuloma venereum associated L-Serovars by TaqMan PCR. BMC Infect Dis; 8: 56
3. Jurstrand M, Falk L, Fredlund H, Lindberg M, Olcén P, *et al.* (2001). Characterization of *Chlamydia trachomatis* *omp*1 genotypes among sexually transmitted disease patients in Sweden. J Clin Microbiol; 39:3915-3919
4. Lutter EI, Bonner C, Holland MJ, et al. (2010). Phylogenetic analysis of *Chlamydia trachomatis* Tarp and correlation with clinical phenotype. Infect Immun. 2010 Sep; 78: 3678-3688.
5. Gomes JP, Bruno WJ, Nunes A, et al. (2007). Evolution of *Chlamydia trachomatis* diversity occurs by widespread interstrain recombination involving hotspots. Genome Res. 2007 Jan; 17: 50-60

Description of specific strains used in the phylogenetic reconstructions shown in this work (Figs. 1-3).

**Table C**. Reference sequences used for phylogenetic analyses.

| ***omp*A genotype** | **Strain** | **Genbank access number** |
| --- | --- | --- |
| D | D_ 14-96 | CP006677 |
|  | D_UW3-CX | AE001273 |
|  | D_SotonD6 | HE601800 |
|  | D_SotonD5 | HE601799 |
|  | D-LC | CP002054 |
|  | D-EC | CP002052 |
|  | D_13-96 | CP006676 |
|  | D_SotonD1 | HE601798 |
|  | D-s2923 | ACFJ01000001 |
| E | E-Bour | EU247655 |
| F | F-SW4 | HE601804 |
| G | G-9301 | CP001930 |
|  | G-9768 | CP001887 |
|  | G-11074 | CP001889 |
|  | G-11222 | CP001888 |
|  | G-SotonG1 | HE601807 |
|  | G_UW57 | AY184162 |
| I/Ia | Ia 20-97 | CP006678 |
|  | IaSotonIa3 | HE601809 |
|  | IaSotonIa1 | HE601808 |
| J/Ja | J_31-98 | CP006680 |
|  | J_6276tet1 | CP002680 |
|  | J_6276s | ABYE01000001 |
|  | J_6276 | ABYD01000001 |
| K | K_SotonK1 | HE601794 |
| L1 | L1-115 | HE601952 |
|  | L1-440 | HE601950 |
| L2/L2b | L2-434 | CO003963 |
|  | L2b-UCH1 | AM884177 |
| L3 | L3-404 | HE601955 |
| A | A-HAR | CP000051 |
| B | B_Jali20_OT | FM872308 |
| C | C_TW-3 | AY184158 |
